# Introduction for special issue on neuroimmune interactions in chronic pain

**DOI:** 10.1097/PR9.0000000000000894

**Published:** 2021-03-09

**Authors:** Marzia Malcangio

**Affiliations:** Wolfson Centre for Age-Related Diseases, King's College London, Guy's Campus, London, United Kingdom

**Keywords:** Neurones, Microglia, Macrophages, Dorsal root ganglia, Spinal cord

## Abstract

The growing awareness of the critical role played by the innate and adaptive immune systems in the mechanisms underlying chronic pain has prompted further research examining the modalities by which immune cells communicate with neurones, the aim being to identify new players involved in inflammatory and neuropathic pain signalling. This collection of research includes 9 articles on neuroimmune interactions as the underlying mechanisms for neuropathic pain including peripheral neuropathies, pain in rheumatoid arthritis and osteoarthritis, and bone cancer pain. The immune cells under scrutiny include macrophages, microglia, osteoclasts, and B cells, and their interactions with neurones in locations such as the dorsal root ganglia, blood–spinal cord barrier, spinal cord, and brain. Our hope is that this body of work may serve to furnish existing interest while also constituting a springboard of sorts for indispensable further investigation on neuroimmune interactions in chronic pain.

## Abstract 2

This body of work may serve to furnish existing interest while also constituting a springboard of sorts for indispensable further investigation on neuroimmune interactions in chronic pain.

## 1. Introduction

Pain is an unpleasant sensory and emotional experience triggered in response to an actual or potential threat to the integrity of the body and therefore constitutes a protective mechanism. The loss of this protective mechanism has considerable physical consequences that interfere with quality of life. However, more commonly, pain outlives its usefulness and becomes chronic pain, which is profoundly different from acute pain as it could stem from plastic changes within the pain pathways.^3,12^

Acute pain perception involves specialised sensory neurones—namely nociceptors—which transmit noxious signalling to dorsal horn neurones in the spinal cord. From here, the transmission ascends to higher centres in the brain. It is in the brain that the intensity and type of pain is perceived, the emotional component is registered, and followed by protective actions in an effort to safeguard the body's integrity.^3^

Chronic pain can be a debilitating condition, in some cases it lasts longer than the initial stimulus and for which the pain experienced is disproportionate to its instigator. Chronic neuropathic pain, such as that in peripheral neuropathy, and chronic musculoskeletal pain, such as that in osteoarthritis and rheumatoid arthritis, can be characterized by an increase both in sensitivity to painful stimuli (hyperalgesia) and in painful response to nonnoxious stimuli (allodynia).

Under sensitised pain conditions local inflammatory reactions—for instance, through macrophage accumulation and cyto (chemo) kine production—facilitate the sensory neurones' activation and contribute to peripheral sensitisation. The increased afferent input into the dorsal horn of the spinal cord, remotely from the injury, causes increased neuronal activity (central sensitisation) and significant responses by resident immune cells—namely microglia—which further promote neurone activity by releasing pronociceptive factors.^9,13^

The growing awareness of the critical role played by the innate and adaptive immune systems in the mechanisms underlying chronic pain has prompted further research examining the modalities by which immune cells communicate with neurones, the aim being to identify new players involved in inflammatory and pathological pain signalling.^6,9^

This collection of research includes 9 articles on neuroimmune interactions as the underlying mechanisms for neuropathic pain including peripheral neuropathies, pain in rheumatoid arthritis and osteoarthritis, and bone cancer pain. The immune cells under scrutiny include macrophages, microglia, osteoclasts, and B cells, and their interactions with neurones in locations such as the dorsal root ganglia (DRG), blood–spinal cord barrier, spinal cord, and brain.

The role of microglial-mediated mechanisms in neuropathic pain (but also morphine-induced hyperalgesia) is thoroughly covered by Khono and Tsuda^7^ within their well-received work on the P_2_X_4_ receptor that is de novo expressed by dorsal horn microglia after peripheral nerve injury. P_2_X_4_ activation by adenosine triphosphate results in the release of brain-derived neurotrophic factor that, through neuronal TrkB receptor activation, generates a decrease in the potassium/chloride transporter and leads to a depolarising shift in chloride ion reversal potential, which ultimately results in gamma aminobutyric acid being less inhibitory. This review offers a critical perspective on the suitability of P_2_X_4_ receptor blockers for the treatment of chronic pain and the role of microglial activation in human chronic pain conditions.

Garcia-Fernandez et al.^4^ introduce the low density lipoprotein receptor-related protein 1 (LRP-1) as playing a potential protective role in neuropathic pain: for instance, the activation of LRP-1 in microglia results in a decrease of proinflammatory cytokine expression. This comprehensive review examines LRP-1 and LRP-1 ligands potentials in the treatment of neuropathies.

Balogh et al.^2^ discuss the role of components within the renin–angiotensin system in neuropathic pain, placing emphasis on the octapeptide angiotensin II (Ang II) and modulators of G-protein-coupled receptors ATR1 and ATR2, which are expressed in pain-related areas of the brain by neurones, microglia, and astrocytes. They explore and evaluate evidence for the efficacy of AT2R antagonists in neuropathic pain preclinical and clinical work. Their own data demonstrate that AT2R activation by Ang II in macrophages contributes to nociceptive hypersensitivity in neuropathic pain models. Macrophages are found in significant numbers at the site of nerve injury, and Ang II facilitates monocyte/macrophage infiltration into said site and triggers production of radical oxygen species that can activate TRPA_1_ receptors on sensory neurones to induce nociceptive signalling.

Meanwhile, Andriessen et al.^1^ explore the mechanisms underlying bone cancer pain, which shares feature with neuropathic and inflammatory pain and results from breast and prostate cancers metastases to the bone, a tissue that is richly innervate by DRG nociceptors. Here, the innate immune cells scrutinised are bone-resorbing osteoclasts, whose differentiation is accelerated by tumour cells and are found in close vicinity to the nociceptor peripheral terminals. Osteoclasts contribute to pain by producing proinflammatory cytokines and chemokines, by creating an acidic extracellular environment, and also indirectly by increasing bone resorption that weakens the bone and leads to nerve compression and fractures. Osteoclastogenesis provides scope for innovative antinociceptive targets that can be combined with the use of bisphosphonates for bone cancer pain relief. Most relevant to this special issue is the mention afforded to inhibitors of colony-stimulating factor 1 receptors (CSF1R) that may be considered for the reduction of pain in bone cancer models on the basis of preclinical evidence.

Indeed, there is convincing evidence reviewed by Yu et al.^14^ that supports the existence of a role for CSF1 and CSF1R in neuropathic pain, with specific attention on sensory neurone-derived CSF1, which is de novo expressed in injured neurones downstream of transcription factor ATF-3. Extracellular CSF1 acts on CSF1R expressed by microglia in the spinal cord and resident macrophages in the DRG and contributes to these cells' acquisition of a pronociceptive phenotype as well as the establishment of neuropathic allodynia. Moreover, CSF1 mediates dorsal horn microglial proliferation after peripheral nerve injury to a significant extent—this is an important contribution that deserves further investigation considering microglia are self-renewal cells and monocytes do not seem to infiltrate the spinal cord after peripheral nerve injury. The latter topic is covered in greater depth by Montague-Cardoso and Malcangio^10^ who examine existing evidence suggestive of a possible breach of the blood–spinal cord barrier in models of neuropathic and inflammatory pain. This breach, when established, generates monocyte/macrophage infiltration and the establishment of pronociceptive communication with dorsal horn neurones alongside the microglia.

Yu et al.^14^ also maintain that DRG tissue macrophages play a significant role in the induction of neuropathic allodynia and display a similar role in both male and female mice. The authors favour the possibility that DRG resident macrophages are a self-maintained population that proliferates after peripheral axon injury. What is more, they discuss a pivotal role for neurone-derived CSF1 in macrophage proliferation in male mice only.

Meanwhile, Silva et al.^11^ are in agreement with the emerging suggestion that DRG resident macrophages are a self-maintained population that proliferates and contributes to neuropathic allodynia after nerve injury and chemotherapy drug treatments. They propose that the activation of toll-like receptors (TLR2, TLR9, and TLR4) and nucleotide-binding oligomerization domain-like receptors in macrophages results in the production of cytokines and radical oxygen species that exacerbate nociceptive signalling. Alongside damage-associated molecular patterns such as the high-mobility group box-1 released by neurones, the suggestion is that pathogen-associated molecular patterns derived from gut microbiota may reach the DRG through the circulation and activate resident macrophages.

The role of macrophages in the pathogenesis of osteoarthritis (OA) joint pain and their communication with nociceptors in the joints is considered by Geraghty et al,^5^ with a view to exploring new avenues towards novel interventional strategies. In this review, we find interesting comparisons of synovial macrophage phenotypes in OA and rheumatoid arthritis (RA) joints in both preclinical and clinical samples. The overall submission is that the profiling of macrophages may predict OA disease severity and pain, and that macrophage-associated pathways may provide a source of targets for OA pain. In animal models of OA, growing evidence indicates that synovial macrophages, DRG macrophages, and microglia in the spinal cord contribute to the underlying pain mechanisms. This comprehensive review article will stimulate more work on neuroimmune interactions in OA pain.

Remaining in the area of RA, with the addition of complex regional pain syndrome and channelopathies from potassium channel complex autoimmunity, Lacagnina et al.^8^ ask how autoantibodies contribute to chronic pain mechanisms in autoimmune diseases. Here, the adaptive immune cells under scrutiny are pathogenic B cells that release cytokines or autoantibodies and interact with the nociceptor peripheral terminals (eg, in the joint synovium) and cell bodies in the DRG. The critical point made in this final review is that pain in autoimmune diseases can be present before clinical signs of inflammation, and emerging evidence indicates that immunoglobulins (IgG and IgM) can promote persistent pain states. IgG and IgM antibodies activate complement components that are pronociceptive through local cytokine production that sensitises nociceptive neurones. In addition, the Fc region of IgG can interact with Fcγ receptors expressed by immune cells (including microglia) and sensory neurones, which, after activation, can promote several events leading to pronociceptive effects.

To conclude, I trust this body of work serves to furnish existing interest while also constituting a springboard of sorts for indispensable further investigation on neuroimmune interactions in chronic pain.

## Disclosures

The author has no conflicts of interest to declare.
